# Modifications of Hydroxyapatite by Gallium and Silver Ions—Physicochemical Characterization, Cytotoxicity and Antibacterial Evaluation

**DOI:** 10.3390/ijms21145006

**Published:** 2020-07-15

**Authors:** Kamil Pajor, Łukasz Pajchel, Anna Zgadzaj, Urszula Piotrowska, Joanna Kolmas

**Affiliations:** 1 Department of Analytical Chemistry, Chair of Analytical Chemistry and Biomaterials, Medical University of Warsaw, Faculty of Pharmacy, ul. Banacha 1, 02-097 Warsaw, Poland; kamil.pajor@wum.edu.pl (K.P.); lukasz.pajchel@wum.edu.pl (Ł.P.); 2 Department of Environmental Health Sciences, Medical University of Warsaw, Faculty of Pharmacy, ul. Banacha 1, 02-097 Warsaw, Poland; anna.zgadzaj@wum.edu.pl; 3 Faculty of Medical Sciences and Health Sciences, Kazimierz Pulaski University of Technology and Humanities in Radom, Chrobrego 27 St., 26-600 Radom, Poland; piotrowska_ula@wp.pl

**Keywords:** hydroxyapatite, gallium, silver, PXRD, NMR, FT-IR spectroscopy, antibacterial activity

## Abstract

Hydroxyapatite (HA) powders enriched with silver or gallium ions or both were synthesized by two different routes: standard precipitation and the solid-state method. The powders were characterized by using several methods: inductively coupled plasma optical emission spectrometry (ICP-OES), powder X-ray diffractometry (PXRD), transmission electron microscopy (TEM), infrared spectroscopy (FT-IR) and solid-state nuclear magnetic resonance spectroscopy (ssNMR). The effects of enrichment of the HAs in Ag^+^ or Ga^3+^ or both on in vitro cytotoxicity and microbiological activity were discussed. PXRD experiments showed that the samples obtained by the wet method consisted of single-phase nanocrystalline HA, while the samples prepared via the solid-state method are microcrystalline with a small amount of calcium oxide. The introduction of higher amounts of silver ions was found to be more effective than enriching HA with small amounts of Ag^+^. Gallium and silver ions were found not to affect the lattice parameters. Ga^3+^ affected the crystallinity of the samples as well as the content of structural hydroxyl groups. Among samples synthesized by the wet method, only one (5Ag-HAw) was cytotoxic, whereas all Ga-containing samples obtained by the dry method showed cytotoxicity. In the preliminary antimicrobial test all the materials containing “foreign” ions showed high antibacterial activity.

## 1. Introduction

Life span has been steadily increasing for many years. As a consequence, the number of orthopaedic surgeries related to filling bone defects, fracture treatment or joint replacement is continually increasing. According to Weiser et al., approximately 313 million surgeries were performed in 2012 in member states of the World Health Organization (WHO), an increase of 38% over the previous eight years [1]. Unfortunately, along with this upsurge in the number of orthopaedic surgeries, a significant increase in surgical site infection (SSI) has also been observed [2,3,4]. SSI is the most common hospital-acquired infection (HAI) in low-income and middle-income countries (LMICs) and the second or third most frequent HAI in high-income countries (HICs) [5,6]. In LMICs, the occurrence of SSI ranges from 6.1–7.8%, and for HICs, this occurrence ranges from approximately 1–3% [7]. Orthopaedic surgeries, such as hip and knee prosthesis, are among the highest cumulative incidences of SSI [5]. SSI may lead to an osseous infection, followed by a systemic infection, which requires the usage of perioperative antibiotic prophylaxis (PAP) to reduce the risk of infection [8,9]. PAP consists of a short-term administration of antibiotics directly before surgery (during the induction of anesthetic before skin incision). However, if infection occurs despite prophylaxis, appropriate antibiotic therapy is then used, where drugs are administered orally or intravenously [2,3,10,11].

Regrettably, due to the weak vascularity of bone tissues, the aforementioned routes of administration enforce the use of high dosages of antibiotics, which can cause serious adverse effects and may destroy the natural microbiota of the human body [9,12]. Notably, the number of drugs suitable for the treatment of bone infections is limited. At the same time, the spectrum of available antibiotics is even smaller when the infection results from resistant strains of bacteria, especially methicillin-resistant *Staphylococcus aureus* (MRSA) [3,12,13]. The apparent problem is the expansion of antibiotic resistance in many bacterial strains [4,12,14,15,16].

Therefore, alternative routes of antibiotic administration (bone drug delivery systems) and other antimicrobial agents (peptide, polymers, metal nanoparticles and ions) have been widely investigated [12,16,17,18]. Delivering antibacterial substances directly into bone tissue allows the reduction of side effects and inconveniences related to the intravenous or oral administration of drugs. Moreover, with such drug carriers, a therapeutic dose of antibiotic can be reduced, and, in particular, the drug can be sustainably released [17].

The above solution can be achieved by using calcium phosphates (CaPs), due to their ability to facilitate antibiotics adsorption [18]. The primary representative of the CaPs group is hydroxyapatite (HA) because of its biocompatibility with bone mineral as well as its physicochemical properties. HA is frequently used in the treatment of several bone diseases. Moreover, synthetic HAs are harnessed, especially in implantology, where HAs are used as scaffolds for newly formed bone. HA may also support bone rebuilding and remodeling [19,20,21,22]. Additionally, HA is characterized by a high ion exchange capacity, which significantly increases its uses in various fields of medicine [19,20,22].

Silver has been widely known for hundreds of years as a highly effective antibacterial agent. Silver’s antimicrobial mechanism is mainly based on the exchange of hydrogen atoms with silver atoms in proteins’ thiolic groups (-SH) [23]. Due to the aforementioned exchange, sulphur-silver (S-Ag) bonds are formed, leading to denaturation and inactivation of proteins located in the cytoplasm and cell membrane, resulting in the dysfunction of membrane pumps. Bacterial cell membrane shrinks and detaches from the cell wall, which leads to its destruction. Silver ions exhibit lower activity against Gram-positive bacteria than Gram-negative bacteria, which can be caused by protective properties of the peptidoglycan layer of G+ bacteria. Unfortunately, silver ions in higher concentrations exhibit high toxicity in human body cells [23,24,25].

Gallium ions are significantly less toxic than silver and have a different antibacterial mechanism, primarily based on the substitution of iron ions in protein metabolism, which leads to the disability of cell functions. Notably, gallium ions show positive effects on bone tissue through the induction of osteoblasts and protection from increased resorption by inhibiting osteoclast activity and modifying crystal solubility, which is harnessed in the treatment of osteoporosis [24,26,27,28,29]. Therefore, simultaneously enriching HA with silver and gallium ions may lead to a material characterized by high antibacterial efficiency and low toxicity to human cells.

In this work, the study was focused on the synthesis of HA materials modified by gallium and silver ions. The materials were obtained by two different methods: a standard wet precipitation and a solid-state method. A comprehensive structural analysis was undertaken to investigate the effect of single and dual introduction of Ag^+^ and Ga^3+^ into the HA materials. Moreover, preliminary microbiological activity and toxicity to human cells of the obtained powders were examined.

## 2. Results

### 2.1. Physicochemical Properties of the Obtained Powders

**Figure 1:**
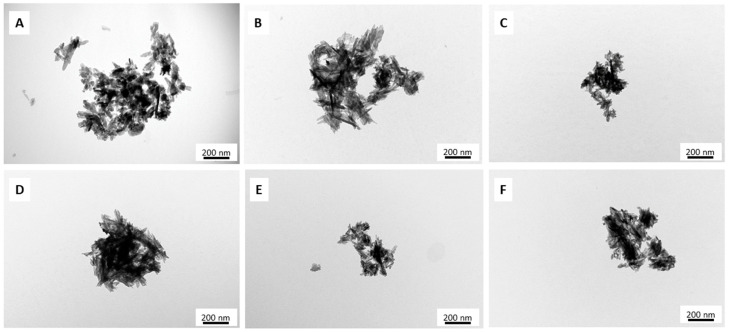
Transmission electron microscopy (TEM) images of the samples: HAw (**A**); 1Ag-HAw (**B**); 5Ag-HAw (**C**); 5Ga-HAw (**D**); 1Ag5Ga-HAw (**E**) and 5Ag5Ga-HAw (**F**).

The TEM micrographs (Figure 1) of the samples synthesized by the wet method revealed that the powders consisting of nanometer-sized crystals had a great tendency to form agglomerates. In most cases, these nanocrystals were characterized by an elongated, rod-like shape and despite the incorporation of silver or gallium ions or both, the morphology of the HAs remained almost unchanged. However, the introduction of silver ions into the HA materials caused a slight reduction in particle size. In turn, the enrichment of the apatitic crystals with gallium ions seemed to affect the nanoparticles’ shapes: they appeared more like thin needles. The powders containing silver and gallium exhibited rod-like and needle-like morphology: the nanoparticles were finer than in the pure HA and HA with only one kind of additive ions.

The morphology of samples synthesized by the dry method is shown on the representative micrograph in Figure S1 (Supplementary Materials). The powders consisted of thick and tight microsized particles that exhibited an irregular plate-like morphology. Notably, long-term heating during synthesis caused an early stage of sintering, with slight densification of particles [30].

The results of the elemental analysis of the obtained samples are presented in Table 1. In the 1Ag-HAw and 5Ag-HAw samples, silver concentration determined by the inductively coupled plasma optical emission spectrometry (ICP-OES) method were 0.06 and 0.46 mass%, which was 56% and 92% of the nominal values, respectively. In turn, in the 1Ag-HAd and 5Ag-HAd samples, silver content was 0.01 and 0.39 mass%, which was 9.3% and 72% of the nominal values, respectively.

Therefore, one can conclude that the introduction of higher amounts of silver is more effective than enriching HA with small amounts of silver. Moreover, the introduction of Ag^+^ ions in the HA materials via a solid-state method was significantly less effective. When introducing gallium ions, the efficiency was markedly different, and in the samples with only one additive, it was close to 100%. In Ag^+^ and Ga^3+^-enriched samples (synthesized via both methods), the gallium content was close to the nominal value, while the silver ion concentration was much lower than the nominal value, as well as its content in Ag^+^-enriched HAs (Table 1). Thus, it may be suggested that the simultaneous introduction of Ag and Ga inhibits silver incorporation.

The overall [Ca site]/[P site] molar ratio was calculated by including silver and/or gallium contents of the samples and presuming the location of Ag and Ga in the Ca sites. Considering that the Ca/P molar ratio in stoichiometric HA was 1.67, samples obtained via the wet method had a [Ca site]/[P site] molar ratio close to the stoichiometric value. In contrast, samples synthesized by the dry method exhibited a [Ca site]/[P site] molar ratio significantly higher than expected (1.76–1.88). These higher values show that the final products contained less phosphate than the reagents’ composition suggests.

**Figure 2:**
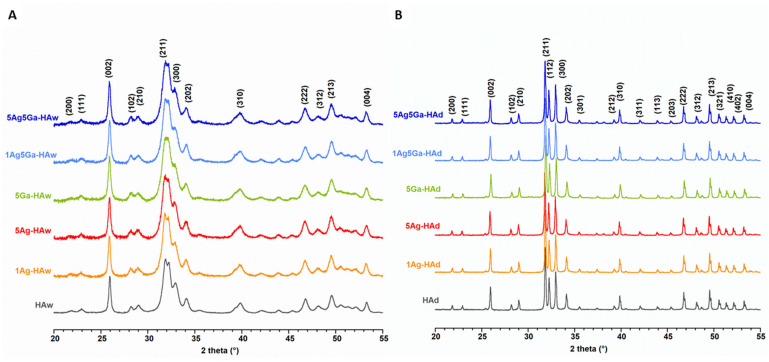
Powder X-ray diffractograms (PXRD) of the samples synthesized by the wet (**A**) and dry (**B**) method.

All diffraction patterns were indexed with the standard, hexagonal structure of HA (JCPDS Np. 09-0432). Representative diffractograms of the samples synthesized by the wet method are presented in Figure 2A. The broad and weakly resolved reflections show that the powders were formed by a pure, homogeneous apatitic phase.

The introduction of ‘foreign’ Ag^+^ and Ga^3+^ ions into the structure of apatite did not affect the position of the reflections nor their relative intensities [31,32]. However, one can observe a slight line broadening of the reflections that was used, based on Scherrer’s formula, to calculate the average crystalline size (Table 2) [33]. The enrichment of HA with silver and gallium resulted in slightly smaller crystals.

The a and c lattice parameters did not show significant differences compared to the parameters of pure HA. These results may suggest the very small amounts of ionic additives that do not affect the unit cell parameters and its size. What is more, the ions introduced into the materials can be located not only in the nanocrystalline core, but also on the hydrated surface layer [30,32]. Our results are consistent with previous studies on HA containing Ga^3+^ ions [32]. Kurtjak et al. suggested that wet synthesis gallium ions are located especially in the amorphous hydrated surface layer of HA. High concentration of gallium ions lead to the development of amorphous layer covering the crystalline apatitic core [32].

PXRD measurements for dry-synthesized samples determined the formation of highly crystalline HA for all materials (Figure 2B). Notably, an additional reflection at approximately 38.13° was detected in the diffractograms of the 5Ag5Ha-HAd and 5Ag-HAd samples, which likely corresponds to calcium oxide that is often a contaminant in HA synthesized at high temperatures. In order to compare the crystallinity of powders obtained by the solid-state method, the formula proposed by Landi et al. [34] was used: *Χ* ≅ 1 − (V_112/300_ − I_300_), where *Χ* is the crystallinity degree, I_300_ is the intensity of (300) peak and V_112/300_ is the intensity of the hollow between (112) and (300) peak. The results showed that samples containing gallium were less crystalline, while the silver content did not affect crystallinity, which may be due to the small concentration of silver ions.

The cell parameters of all the samples are presented in Table 2, which shows that there were no significant differences between them. We expected the changes due to the substitution of larger-size Ag^+^ (129 pm) and smaller-size Ga^3+^ (76 pm) for Ca^2+^ (114 pm) cations [35]. However, we could assume that low concentration of silver and gallium ions had not affected the lattice parameters and its sizes.

**Figure 3:**
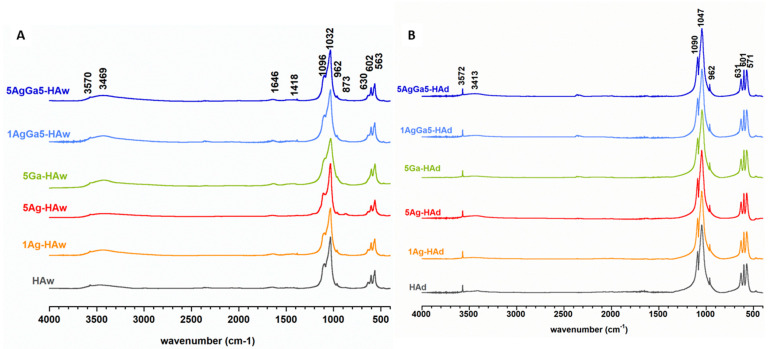
FT-IR spectra of the samples synthesized by the wet (**A**) and dry (**B**) method.

The FT-IR spectra of all synthesized products are presented in Figure 3A,B. In the spectra of materials synthesized via two different methods (wet synthesis and solid-state synthesis), several distinctions were evident. Generally, the bands in the spectra of dry-synthesized samples were sharper and significantly better resolved, which indicates, in great accordance with PXRD results, their well-ordered crystal structure and better crystallinity.

The spectra mainly consisted of intensive ν_1+_ν_3_ phosphate bands in the 1100–960 cm^−1^ region and ν_4_ phosphate bands in the 602–560 cm^−1^ region [3]. In the spectra of samples synthesized by the wet method with the phosphate bands, the presence of adsorbed water was detected, with bands at approximately 3469 (stretching) and 1646 cm^−1^ (bending) [36,37,38,39]. These bands were invisible, or at least, quite small, in the spectra of the dry-synthesized samples. In the spectra of the wet-synthesized samples, a single, sharp band occurred at 1384 cm^−1^, which may be attributed to residual nitrates after precipitation. Thus, all samples from both methods produced the characteristic structural OH groups stretching and librational bands visible at approximately 3570–3572 cm^−1^ and 630–631 cm^−1^, respectively. Upon thorough visual analysis, the relative intensity of these bands differed in the obtained spectra. Therefore, a curve-fitting analysis of the 650–500 cm^−1^ region was performed (Figure S2).

The results presented in Table S1 revealed a reduction of the relative intensity of the librational OH band in samples containing foreign ions, especially gallium. A reliable explanation of this phenomenon is not trivial. According to available reports, the content of structural hydroxyl groups depends on the crystallinity of HA, the presence of some foreign ions in the HA crystal structure and on the size of crystals [40,41,42]. Usually, nanocrystals of apatite are OH-deficient as compared to stoichiometric, well-crystallized HA. High affinity of HA for ionic substitution means that during the wet synthesis carbonate ions can be easily substituted for orthophosphates in the crystal lattice. Then, to maintain the charge balance, calcium and hydroxyl ions are simultaneously released [41,42]. However, samples synthesized by the dry method were microcrystalline with well-ordered crystals. FT-IR spectra indicate the absence of carbonate in these samples. Moreover, the theoretical mechanism of the substitution of gallium into the apatitic crystal is quite complicated: gallium (charged +3) was substituted for calcium (charged +2). Therefore, during substitution, the differences in charges should be balanced. Admittedly, gallium ions do not substitute in calcium cations; they only adsorb on the crystal surface or enter on interstitial positions [43,44]. Our research on unit cell parameters demonstrated no significant changes in gallium-containing samples, but we observed reduction of crystallinity. Thus, according to the literature [40,41,43,45] we may suppose that the structural OH groups reduction may primarily be affected by an uptake of structural water into the c-axis channel.

**Figure 4:**
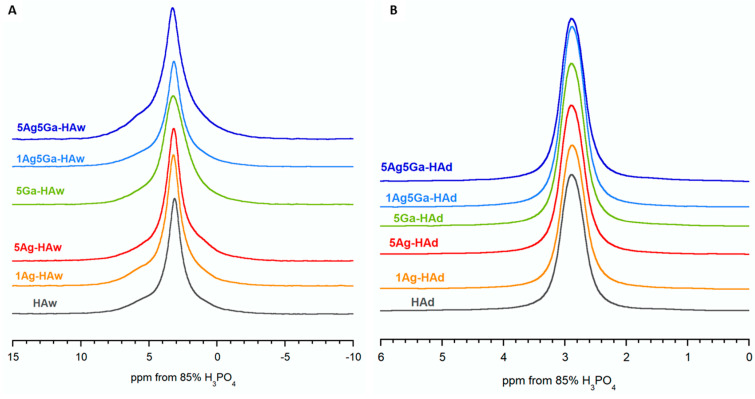
^31^P cross-polarization magic angle spinning nuclear magnetic resonance (^31^P CP MAS NMR) spectra of the samples synthesized by the wet (**A**) and dry (**B**) method.

Figure 4 shows the ^31^P cross-polarization (CP)/magic angle spinning (MAS) NMR spectra of the powders obtained by the wet and dry methods. All spectra revealed one intensive resonance line at approximately 2.80–3.30 ppm, which is a particular feature of hydroxyapatite [46,47]. This signal was significantly broader in the spectra of wet-synthesized samples (Figure 4A). Moreover, using a line fitting process, it was possible to distinguish two overlapped signals: a narrow one and a broad one (Figure S3 and Table S2). According to the researchers’ previous studies, the narrow line originates from ^31^P nuclei located close to the protons of structural hydroxyl groups of the crystal channels, while the broad line originated from the ^31^P nuclei located close to adsorbed water molecules in the hydrated surface layer [45,48]. This explanation accounted for the lack of a broad line in the spectra of the dry-synthesized samples: they were practically water-free.

Additionally, the width of the analyzed signals from the full-width half-maximum (FWHM) parameter was examined (Table S2). First, samples synthesized by the dry method gave a much narrower signal than wet-synthesized samples, and the FWHM of signals from samples enriched with ‘foreign’ ions was almost always higher than signals from the unsubstituted samples. Since the width of solid-state nuclear magnetic response signals was closely related to crystallinity and the order of the structure, it can be assumed that the dry-synthesized powders were well-ordered and highly crystalline. Moreover, the substitution of gallium and silver affected the crystallinity, which confirmed our previous results from the PXRD and FT-IR methods.

**Figure 5:**
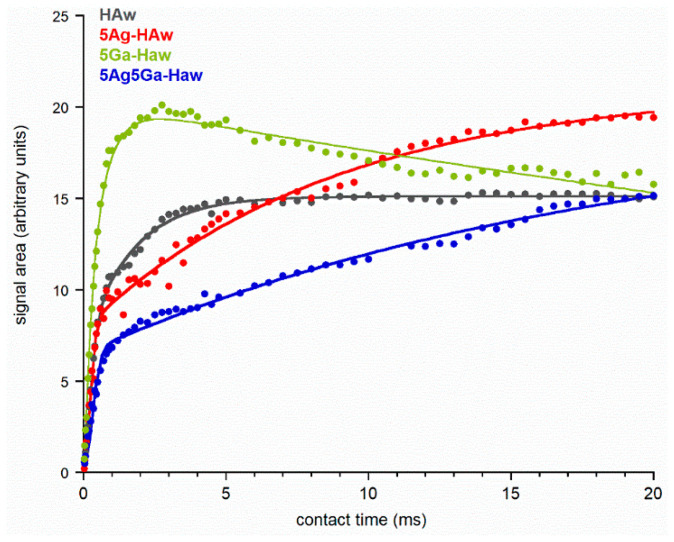
^1^H→^31^P cross-polarization nuclear magnetic resonance (^1^H→^31^P CP NMR) kinetic curves of the representative samples.

In order to provide further structural information, ^1^H→^31^P CP/MAS NMR experiments were performed with variable contact time for selected samples (Figure 5). Then, the dependence of the intensity of the signal (expressed as the peak area) on contact time was investigated. For this analysis, we selected the narrow component lines from the cross-polarization (CP) spectra of the samples obtained by the wet method. Clearly, the obtained curves significantly differed; however, they all follow a non-classical NMR kinetic model [49], which is described by a physical function:

I(t) = *I*_0_*exp* (−*T_1ρ_^H^*)[1 − *λexp*(−*t*/*T_df_*) − (1 − *λ*) *exp*(−1.5*t*/*T_df_*) *exp* (−0.5*t*^2^/*T*_2_^2^)], (1)

where *I_0_*—a signal amplitude; *T_1ρ_^H^*—a proton spin-lattice relaxation time in the rotating frame; *T_df_*—a time constant of proton spin diffusion; *T_2_*—a CP time constant in the non-classical CP model (1/T_CP*_ is the CP rate), which characterizes the polarization transfer;

*Λ*—a parameter specific to a cluster of ^1^H and ^31^P nuclei involved in initial CP; for a rigid lattice *λ* = (*n* + 1)^−1^, where *n* is the number of protons close to the observed ^31^P nucleus; however, *λ* is dependent on molecular motion, and as such should be treated as an adjustable parameter.

Generally, kinetic curves illustrate three stages. In the first stage, the CP process begins in ^1^H and ^31^P spin clusters. In all our cases, the signal intensity increased very quickly (with a similar time constant of *T_2_*, see Table 3). Then, subsequent spin diffusion with the remaining bulk protons allowed the CP process to continue. The second stage was described by the constant *T_df_* (diffusion time), and the increase of the signal intensity was much slower. Notably, the higher the value of *T_df_*, the slower the spin diffusion between protons occurs. Thus, in the 5Ag5Ga-HAw sample, the spin diffusion was significantly slower than in the HAw sample, which may indicate a much lower proton density in the substituted sample.

The final stage is related to proton spin-lattice relaxation and is described by the time constant *T_1ρ_^H^*. For HAw, 5Ag-HAw, and 5Ag5Ga-HAw samples, the kinetic curves were characterized by an infinitely long *T_1ρ_^H^* (the curves steadily rose or had reached a plateau). Only for the 5Ga-HAw sample, the signal intensity gradually decreased for long contact times. Thus, in all the cases, polarization occurred in the protons of hydroxyl groups from the crystal lattice, characterized by a very long *T_1ρ_^H^* time.

### 2.2. Biological Tests

The cytotoxic effect of the obtained samples was measured using a BALB/c 3T3 clone A31 mammalian cell line. The results are presented in Table 4 and Table S3.

As expected, the cytotoxicity test showed that the “pure” samples (HAw and HAd) were non-toxic and did not cause harmful extractable effects under the conditions used in this test. Among samples synthesized by the wet method, only one (5Ag-HAw) was cytotoxic. We could conclude that this was due to the highest silver content in this sample (according to results obtained by the ICP-OES method, shown in Table 1). However, the cytotoxicity of this sample significantly decreased with serial dilutions of the extract. Only the undiluted sample was highly cytotoxic (100 mg/mL with 0% of viable cells), while the rest of dilutions did not affect the cell culture condition negatively.

Moreover, it is worth noting that the 5Ga-HAw sample was found to be non-cytotoxic, whereas all samples containing gallium and obtained by the dry method revealed cytotoxicity. The most cytotoxic was sample 5Ga-HAd (IC50 < 12.5 mg/mL). Exposition to the three highest dilutions of extracts prepared with this sample (100–25 mg/mL) resulted in a death of all treated cells. The samples 1Ag5Ga-HAd and 5Ag5Ga-HAd were also cytotoxic, with similar IC50 values (19 and 20 mg/mL, respectively). On the other hand, the samples enriched only in silver (1Ag-HAd and 5Ag-HAd) were non-cytotoxic. This is an unexpected result due to the fact that Ag^+^ ions are much more toxic than Ga^3+^ [50,51,52]. Understanding this problem requires further research. The investigations should be repeated using both different cell lines and different test conditions. Since this research was based on extracts prepared from the obtained powders, it was crucial to consider the differences in solubility of the obtained samples.

In this work we investigated the synergistic effect of the Ga^3+^ and Ag^+^ ions on the antimicrobial activities of new apatitic biomaterials. The antibacterial activity of the Ga(III) and Ag (I) containing hydroxyapatite was evaluated against the Gram-negative *Pseudomonas fluorescens* strain. *Pseudomonas* are iron-dependent bacteria that produce the water-soluble, yellow-green fluorescent dye, pyoverdine in iron-deficient conditions [53]. *Pseudomonas* are also often associated with acute opportunistic infections [54,55,56].

**Figure 6:**
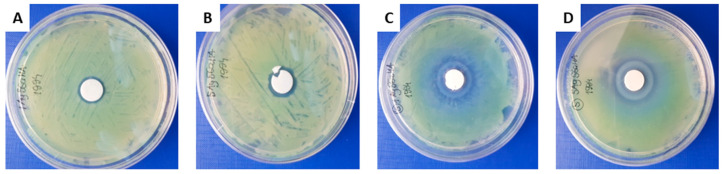
Disc diffusion antibacterial test against *Pseudomonas* *fluorescens* (1Ag5Ga-HAw (**A**); 5Ag5Ga-HAw (**B**); 1Ag5GaHAd (**C**) and 5Ag5Ga-HAd (**D**).

All the tested samples containing “foreign” ions created an inhibition zones (Figure 6 and Table S4). It indicates that enrichment of hydroxyapatites in silver or gallium ions or both provide to obtain biomaterials with antimicrobial activity against *P. fluorescens*. The control HAd and HAw samples created no inhibition zone.

It should be noted that the addition of Ga(III) ions in the HA samples prepared by the solid-state method revealed the presence of the c.a. 40 mm zone of the lack of the fluorescent dye, probably due to the formation of pyoverdine-gallium complexes. Moreover in case of biomaterials synthesized by the solid-state method, the formation of two concentric zones with a total diameter of 27–30 mm were detectable, which could be a result of different diffusion rates of tablet components into the agar [57]. This effect also arose with the HAd sample. Interestingly, in the case of wet synthesis, no effect of pyoverdine-gallium complexing was observed and clear, single growth inhibition zones were shown. The growth inhibition zone in the case of 1Ag5Ga-HAw and 5Ag5Ga-HAw samples increased from 17 to 19 mm together with the increase of silver ion content (Figure 6).

## 3. Materials and Methods

### 3.1. Samples Preparation

Powders of HA, enriched with either Ag^+^ or Ga^3+^ or co-doped with both Ag^+^ and Ga^3+^, were prepared using two different methods: the wet method (simple coprecipitation in an aqueous solution) and the dry method (heating a mixture of reactants). In the wet synthesis, analytical- grade calcium nitrate tetrahydrate (Ca(NO_3_)_2_·4H_2_O, Eurochem BGD, Tarnów, Poland), ammonium dibasic phosphate (NH_4_)_2_HPO_4_, Sigma-Aldrich, St. Louis, MO, USA), silver nitrate (AgNO_3_, Avantor Performance Materials, Gliwice, Poland) and gallium nitrate trihydrate (Ga(NO_3_)_3_·3H_2_O, Sigma-Aldrich, USA) were selected as sources of calcium, phosphorus, silver and gallium, respectively. Calcium carbonate (CaCO_3_; Avantor Performance Materials, Poland), (NH_4_)_2_HPO_4_, silver carbonate (Ag_2_CO_3_) and gallium oxide (Ga_2_O_3_) purchased from Sigma-Aldrich, USA, were used as precursors for dry syntheses. All reagents were distributed so that the [Ca+ (Ag)+ (Ga)]/P molar ratio was approximately 1.67.

In the precipitation method, reagents were dissolved in distilled water. Then, a solution of (NH_4_)_2_HPO_4_ was slowly added to a solution of Ca(NO_3_)_2_·4H_2_O (and optionally, AgNO_3_ and/or Ga(NO_3_)_3_∙3H_2_O) with vigorous stirring. During stirring, an ammonia solution was added drop by drop until the mixture had a pH value of 10. Next, the mixture was heated to 70 °C, stirred for two hours with a magnetic stirrer, and was left to age for 24 h. Finally, the obtained product was filtered, washed several times with distilled water and dried at 100 °C for 24 h.

In the dry method, all starting materials were added to a milling container, with steel balls that served as grinding media. Afterwards, the container was tightly closed and placed in the ball mill where reagents were ground for 30 min. The obtained mixture was then formed into a tablet and heated in an electric furnace at 1100 °C for 24 h.

Each method, wet (w) and dry (d), was used to obtain six different powders:

- Pure, unsubstituted HA (HAw or HAd);
- Ag-containing HA (1Ag-HAw or 1Ag-HAd) with a nominal Ag amount of 0.11 mass% (0.01 mol);
- Ag-containing HA (5Ag-HAw or 5Ag-HAd) with a nominal Ag amount of 0.54 mass% (0.05 mol);
- Ga-containing HA (5Ga-HAw or 5Ga-HAd) with a nominal Ga amount of 0.35 mass% (0.05 mol);
- Ag and Ga containing HA (1Ag5Ga-HAw or 1Ag5Ga-HAd) with a nominal Ag and Ga amount of 0.11 mass% and 0.35 mass%, respectively (0.01 and 0.05 mol, respectively);
- Ag and Ga containing HA (5Ag5Ga-HAw or 5Ag5Ga-HAd) with a nominal Ag and Ga amount of 0.54 mass% and 0.35 mass%, respectively (0.05 mol).

### 3.2. Physicochemical Characterisation

For transmission electron microscopy (TEM), powder samples were immersed in 96% ethanol (Chempur, Piekary Śląskie, Poland). Next, several drops of the obtained suspension were added with a Pasteur pipette to a copper grid, covered with Formvar film and then air-dried. TEM analyses were performed at an accelerating voltage of 80 kV using the JEM-1400 series 120 kV Transmission Electron Microscope (JEOL, Tokyo, Japan), equipped with an 11 Megapixel MORADA G2 TEM camera (EMSIS GmbH, Muenster, Germany).

Inductively coupled plasma optical emission spectrometry (ICP-OES) experiments (elemental analyses) were carried out with the iCAP 7400 Duo Spectrometer (Thermo Scientific, Waltham, MA, USA). In order to prepare HA powders for experimentation, samples were dissolved in a high-purity nitric acid solution (65% HNO_3_) (Merck, Darmstadt, Germany) and then appropriately diluted with deionized water (Milli-Q, Merck).

Phase analyses of the powders were conducted using an X-ray diffractometer (Bruker DX 8 Discover, Karlsruhe, Germany) with CuKα radiation (λ = 1.5406 nm), 40 kV and 40 mA. Powder X-ray diffraction (PXRD) patterns and results were collected in the 2 theta range of 20–70°, with a step size of 0.024°, a step time of 4 s and a locked coupled (theta-theta geometry). Both *a* and *c* lattice parameters of the unit cell, as well as crystallite sizes, were determined using a software for profile and structure analysis (TOPAS, version 3, Bruker). This program uses Rietveld refinement based on analytical profile functions and least squares algorithms to fit a theoretical to a measured XRD pattern. However, we did not perform a full refinement due to the weak resolution of the patterns of wet synthesized samples. The powders were also examined by Fourier transform infrared spectroscopy (FT-IR) using the standard transmission KBr pellet technique (Perkin Elmer Spectrum 1000, Llantrisant, UK). Measurements were performed over a range of 4000–400 cm^−1^, with a 2 cm^−1^ resolution at 30 scans.

Solid-state nuclear magnetic resonance (ssNMR) experiments were carried out using the AVANCE 400 WB spectrometer (Bruker,), operating at 9.4 T. All spectra were recorded at room temperature under magic angle spinning (MAS) at 7 kHz at 32 scans. The ^31^P CP/MAS NMR spectra were obtained with a π/2 pulse of 2.7 μs, a contact time of 2 ms, and a repetition time of 10 s. Additionally, for the selected samples, variable contact time ^31^P CP/MAS NMR experiments were performed with an optimized recycle delay of 10 s and 64 array contact time values ranging from 25 μs to 20 ms.

To process the FT-IR and NMR spectra, GRAMS/AI 8.0 (Thermo Scientific 2008) and NUTS Software (Acorn NMR Inc., Livermore, CA, USA) were used, respectively. Graphics were prepared using KaleidaGraph 3.5 software (Synergy Software, Reading, PA, USA).

### 3.3. Biological Tests

Cytotoxicity of the powders was measured with a neutral red uptake (RNU) test, according to ISO guideline Annex A, with a BALB/c 3T3 clone A31 mammalian cell line [58]. Briefly, BALB/c 3T3 cells were seeded in 96-well microplates containing Dulbecco’s modified Eagle’s medium (DMEM; with a 10% addition of bovine calf serum and two antibiotics: 0.1 mg/mL streptomycin and 100 IU/mL penicillin). Then, cells were incubated for 24 h in strictly defined conditions (37 °C, 5% CO_2_, and humidity higher than 90%). In order to obtain extracts from the powders, samples were incubated for 24 h in a cell culture medium with reduced serum concentration (at 37 °C). Then, the extracts were used to replace a culture medium of BALB/c 3T3 cells. Cells were exposed to various dilutions of each extract in a twofold dilution series for 24 h. Afterwards, extract medium was removed, and cells were washed with phosphate-buffered saline (PBS) and treated with neutral red medium for 2 h. Once again, extract medium was disposed, and cells were washed with PBS and exposed to a desorbing fixative (ethanol and acetic acid aqueous solution). The quantity of neutral red dye accumulated in lysosomes of living cells was colorimetrically assessed at 540 nm. Latex and polyethylene foil were used as positive and negative controls, respectively. Finally, the percentage of viable cells was evaluated by comparing colorimetric analysis results with control group results (untreated BALB/c 3T3 cells incubated in the same conditions as the experimental group). Examined material was considered as non-cytotoxic in a certain range of concentrations if cell viability was not reduced below 70%.

The in vitro antibacterial effect of the hydroxyapatites was performed against the *Pseudomonas fluorescens* PCM 1994 strain from the collection of the Institute of Immunology and Experimental Therapy, Polish Academy of Sciences. The samples of each material were examined using the disc diffusion method. The analyzed powders were uniaxially pressed into pellets (φ = 13 mm) and next heated at 180 °C for 3–4 h in air according to our previous paper [56]. Microbial suspensions of 1.5 × 10^8^ CFU/mL corresponding to 0.5 McFarland density obtained from an overnight culture of *P. fluorescens* developed on TSB were spread onto Petri dishes containing Mueller-Hinton II Agar. After 15 min of preincubation, each material sample was placed onto each Petri discs and then incubated for the next 16–18 h at 37 °C. The diameter of the inhibition zones was measured using a caliper.

## 4. Conclusions

In conclusion, new HA materials containing small amounts of gallium and silver ions were successfully obtained via simple precipitation and solid-state reactions. The physicochemical characterization clearly revealed the formation of nano- and microcrystals in a wet and a dry synthesis, respectively. The efficacy of the enrichment of HA in gallium and silver ions was influenced by the way of synthesis: gallium ions were more effectively introduced in the dry method, while silver ions, inversely, in the precipitation method. Gallium ions in wet synthesized samples seemed to locate on the hydrated surface layer and to lead to the development of amorphous surface layer. The additive ions were introduced into the materials in low concentrations and did not significantly affect the lattice parameters. Among samples synthesized by the wet method, only one (5Ag-HAw) was cytotoxic, whereas all Ga-containing samples obtained by the dry method revealed cytotoxicity.

The antibacterial activity against *P. fluorescens* was evaluated for all the samples. The materials containing silver and gallium ions, even with a small concentration of “foreign ions” revealed a high antibacterial activity.

Future work to be undertaken by the authors will be focused on ions release studies and solubility tests. More extensive studies of the biological activity are in progress, which should give more information.

## Figures and Tables

**Table 1:** Quantitative analysis of silver and gallium and calculated molar [Ca+Ag+Ga]/P ratio from inductively coupled plasma optical emission spectrometry (ICP-OES) data.

	Ag Content mol (mass%)	Efficiency (%)	Ga Content mol (mass%)	Efficiency (%)	[Ca+(Ag)+(Ga)]/P Molar Ratio
HAw	-	-	-	-	1.65 ± 0.03
1Ag-HAw	0.0056 (0.060)	56	-	-	1.67 ± 0.03
5Ag-HAw	0.0460 (0.460)	92	-	-	1.63 ± 0.03
5Ga-HAw	-	-	0.047 (0.33)	94	1.59 ± 0.03
1Ag5Ga5-HAw	0.0037 (0.04)	37	0.046 (0.32)	92	1.64 ± 0.04
5Ag5Ga-HAw	0.0150 (0.16)	30	0.046 (0.32)	92	1.66 ± 0.02
HAd	-	-	-	-	1.70 ± 0.02
1Ag-HAd	0.00093 (0.01)	9.3	-	-	1.85 ± 0.02
5Ag-HAd	0.0360 (0.39)	72	-	-	1.83 ± 0.03
5Ga-HAd	-	-	0.047 (0.33)	94	1.88 ± 0.04
1Ag5Ga-HAd	0.00093 (0.01)	9.3	0.044 (0.31)	88	1.87 ± 0.03
5Ag5Ga-HAd	0.031 (0.33)	62	0.044 (0.31)	99	1.78 ± 0.03

**Table 2:** Various parameters obtained from PXRD data (* Error ± 0.3%).

	Lattice Parameters (Å) *		Crystal Size (nm)		Crystallinity Degree
	*a*-axis	*c*-axis	(002)	(300)	
HAw	9.4113	6.8700	36 ± 2	14 ± 2	0.54 ± 0.03
1Ag-HAw	9.4309	6.8784	34 ± 3	10 ± 3	0.44 ± 0.05
5Ag-HAw	9.4271	6.8783	30 ± 3	7 ± 2	0.46 ± 0.04
5Ga-HAw	9.4222	6.8765	26 ± 2	7 ± 2	0.33 ± 0.04
1Ag5Ga5-HAw	9.4222	6.8765	29 ± 2	8 ± 2	0.40 ± 0.03
5Ag5Ga-HAw	9.4152	6.8782	24 ± 2	8 ± 3	0.37 ± 0.02
HAd	9.4112	6.8754	-	-	0.98 ± 0.02
1Ag-HAd	9.4113	6.8754	-	-	0.97 ± 0.03
5Ag-HAd	9.4203	6.8809	-	-	0.95 ± 0.04
5Ga-HAd	9.3991	6.8677	-	-	0.90 ± 0.02
1Ag5Ga-HAd	9.4146	6.8777	-	-	0.94 ± 0.02
5Ag5Ga-HAd	9.4152	6.8782	-	-	0.92 ± 0.03

**Table 3:** ^1^H→^31^P CP/MAS NMR kinetics parameters for the selected samples.

		HAw	5Ag-HAw	5Ga-HAw	5Ag5Ga-HAw
**PARAMETERS**	*I_0_*	15.1 ± 0.1	21.1 ± 0.4	20.3 ± 0.2	19.2 ± 0.5
	*λ*	0.55 ± 0.02	0.63 ± 0.01	0.70 ± 0.05	0.67 ± 0.02
	*T_df_* (ms)	1.64 ± 0.04	8.8 ± 0.7	0.58 ± 0.03	18 ± 2
	*T_1ρ_^H^* (ms)	∞	∞	71 ± 4	∞
	*T_2_* (ms)	0.27 ± 0.01	0.21 ±0.01	0.29 ± 0.03	0.29 ± 0.01

**Table 4:** Cytotoxicity of the obtained samples (C—cytotoxic; N—non cytotoxic).

Wet Method	Cytotoxicity	Dry Method	Cytotoxicity
HAw	N	HAd	N
1Ag-HAw	N	1Ag-HAd	N
5Ag-HAw	C ^1^	5Ag-HAd	N
5Ga-HAw	N	5Ga-HAd	C ^2^
1Ag5Ga5-HAw	N	1Ag5Ga-HAd	C ^3^
5Ag5Ga-HAw	N	5Ag5Ga-HAd	C ^4^

^1^ IC50 = 60 mg/mL; ^2^ IC50 < 12.5 mg/mL; ^3^ IC50 = 19 mg/mL; ^4^ IC50 = 20 mg/mL.

## Supplementary Materials

The following are available online at https://www.mdpi.com/1422-0067/21/14/5006/s1, Figure S1. TEM micrographs of the 5Ag-HAd (A) and 5Ag5Ga-HAd (B) samples. Figure S2. Curve fitting for the FT-IR spectrum of the 5Ag5Ga-HAw sample. Figure S3. Line fitting for the 31P CP/MAS NMR spectrum of the 5Ag-HAw sample (blue—an experimental line; red—a narrow line; green—w broad line). Table S1. Share of librational bands in the region 700-480 cm ^−1^. Table S2. Curve fitting results for the 31P CP NMR spectra (MAS at 7.0 kHz, the CP contact time of 2 ms). Table S3. Neutral red uptake assay results for all dilutions of extracts. Table S4. Inhibition zone measurements (antimicrobial activity against *P. fluorescens*) obtained from tested samples after incubation in the culture medium.
