# Supplementary material for: Modifications of Hydroxyapatite by Gallium and Silver Ions—Physicochemical Characterization, Cytotoxicity and Antibacterial Evaluation
Source: Int J Mol Sci. 2020 Jul 15;21(14):5006. doi: 10.3390/ijms21145006 (PMC7404191; doi:10.3390/ijms21145006)
Supplement: Supplementary file 1 [file ijms-21-05006-s001.pdf]

International Journal of
Molecular Sciences

MDPI

Supplementary Materials

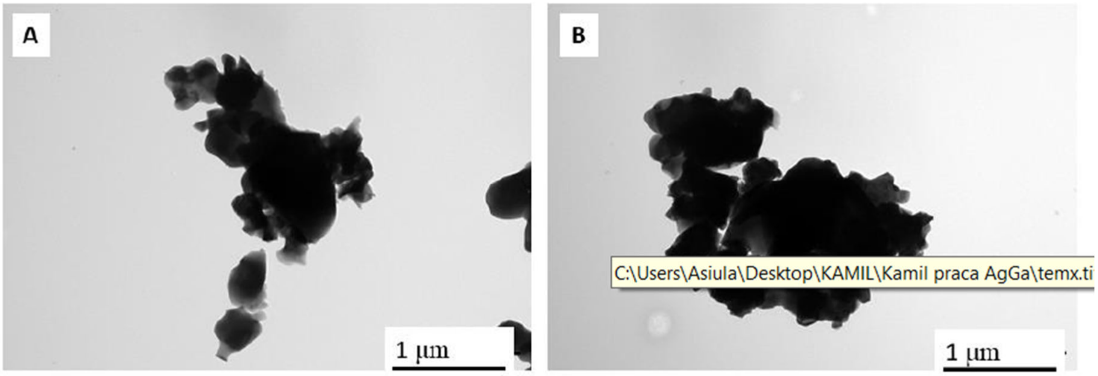

Figure S1. TEM micrographs of the 5Ag-HAd (**A**) and 5Ag5Ga-HAd (**B**) samples.

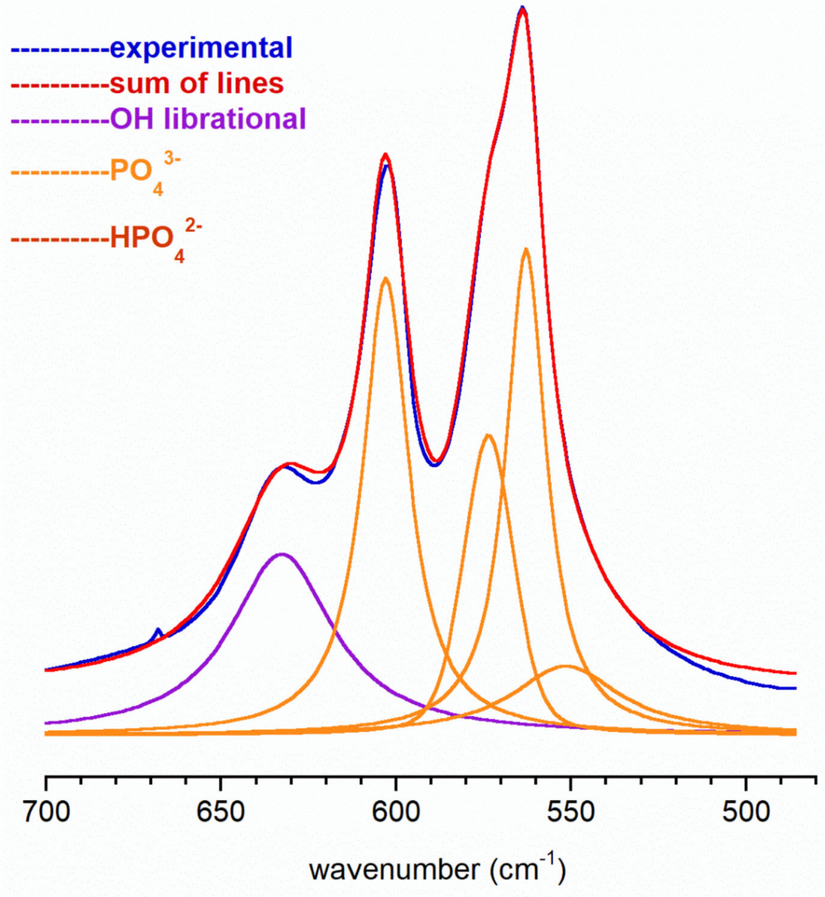

Figure S2. Curve fitting for the FT-IR spectrum of the 5Ag5Ga-HAw sample.

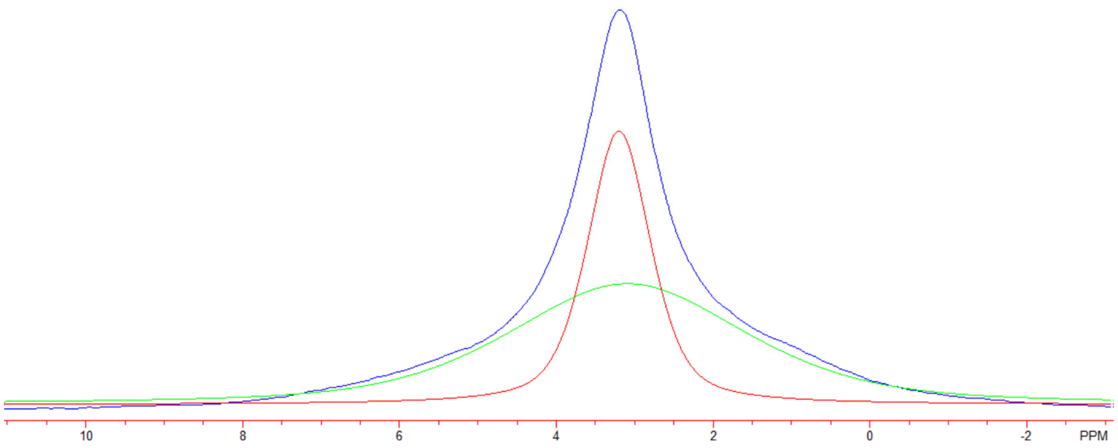

Figure S3. Line fitting for the 31P CP MAS NMR spectrum of the 5Ag-HAw sample (blue – an experimental line; red – a narrow line; green – w broad line).

Table S1. Share of librational bands in the region 700–480 cm^{-1}.

	**% of total area OH librational bands**
HAw	30 ± 3
1Ag-HA	26 ± 2
5Ag-HAw	27 ± 2
5Ga-HAw	23 ± 2
1Ag5Ga-Haw	25 ± 1
5Ag5Ga-HAw	20 ± 2
HAd	34 ± 2
1Ag-HAd	30 ± 3
5Ag-HAd	28 ± 2
5Ga-HAd	21 ± 2
1Ag5Ga-Had	24 ± 2
5Ag5Ga-HAd	20 ± 3

Table S2. Curve fitting results for the ^{31}P CP NMR spectra (MAS at 7.0 kHz, the CP contact time of 2 ms).

	**Chemical shift (ppm)**		**FWHM[a] (Hz)**		**% of total area**	
	**narrow**	**broad**	**narrow**	**broad**	**narrow**	**broad**
**HAw**	3.09	3.01	144	418	51	49
**1Ag-HAw**	3.21	3.31	151	569	38	62
**5Ag-HAw**	3.20	3.10	153	579	38	62
**5Ga-HAw**	3.16	2.95	165	605	31	69
**1Ag5Ga-Haw**	3.17	3.25	154	570	36	64
**5Ag5Ga-HAw**	3.24	3.30	167	618	28	72
**HAd**	2.92	-	71	-	100	-
**1Ag-HAd**	2.87	-	70	-	100	-
**5Ag-HAd**	2.90	-	72	-	100	-
**5Ga-HAd**	2.88	-	74	-	100	-
**1Ag5Ga-Had**	2.88	-	75	-	100	-
**5Ag5Ga-HAd**	2.90	-	78	-	100	-

[a] full width in half minimum (in Hz).

Table S3. Neutral red uptake assay results for all dilutions of extracts obtained from tested samples after incubation in the culture medium.

**Extract concentrations [mg/mL]:**	**100**	**50**	**25**	**12.5**
**Sample:**		**Cells viability [%]:**		
HA-w	89 ± 8	100 ± 4	102 ± 5	103 ± 5
1Ag-HAw	84 ± 4	100 ± 8	97 ± 3	99 ± 5
5Ag-HAw	0 ± 0	92 ± 1	94 ± 2	94 ± 7
5Ga-HAw	82 ± 7	97 ± 9	98 ± 5	102 ± 6
1Ag5Ga-HAW	83 ± 5	89 ± 1	94 ± 3	104 ± 17
5Ag5G-HAW	96 ± 3	108 ± 5	105 ± 0	107 ± 4
HAd	97 ± 10	99 ± 4	101 ± 4	103 ± 3
1Ag-HAd	82 ± 6	93 ± 3	103 ± 3	100 ± 2
5Ag-HAd	81 ± 6	96 ± 2	109 ± 7	105 ± 3
5Ga-HAd	1 ± 1	3 ± 2	1 ± 1	25 ± 8
1Ag5Ga-HAd	4 ± 10	0 ± 1	32 ± 14	75 ± 9
5Ag5Ga-HAd	0 ± 0	4 ± 7	34 ± 8	82 ± 10

Table S4. Inhibition zone measurements (antimicrobial activity against *P. fluorescens*).

**Sample**	**Inhibition zone (mm)**
Haw	13
1Ag-Haw	16
5Ag-Haw	17
5Ga-Haw	17
1Ag5Ga-Haw	17
5Ag5Ga-Haw	19
HAd	13
1Ag-Had	16
5Ag-Had	17
5Ga-Had	27
1Ag5Ga-Had	28
5Ag5Ga-HAd	30

© 2020 by the authors. Licensee MDPI, Basel, Switzerland. This article is an open access article distributed under the terms and conditions of the Creative Commons Attribution (CC BY) license (http://creativecommons.org/licenses/by/4.0/).
